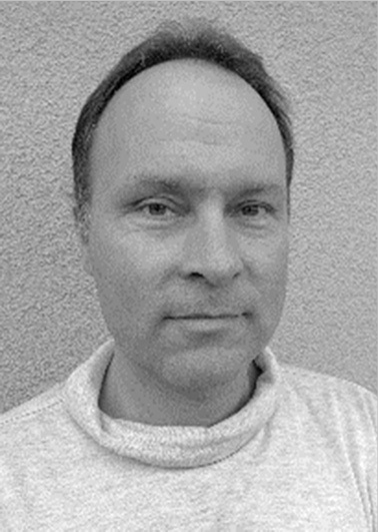# Investigating Protein–Ligand Interactions by Solution Nuclear Magnetic Resonance Spectroscopy

**DOI:** 10.1002/cphc.201701253

**Published:** 2018-02-16

**Authors:** Walter Becker, Krishna Chaitanya Bhattiprolu, Nina Gubensäk, Klaus Zangger

**Affiliations:** ^1^ Institute of Chemistry University of Graz Heinrichstrasse 28 A-8010 Graz Austria

**Keywords:** chemical shift mapping, nuclear magnetic resonance, nuclear Overhauser effect, protein–ligand interactions, saturation-transfer difference

## Abstract

Protein–ligand interactions are of fundamental importance in almost all processes in living organisms. The ligands comprise small molecules, drugs or biological macromolecules and their interaction strength varies over several orders of magnitude. Solution NMR spectroscopy offers a large repertoire of techniques to study such complexes. Here, we give an overview of the different NMR approaches available. The information they provide ranges from the simple information about the presence of binding or epitope mapping to the complete 3 D structure of the complex. NMR spectroscopy is particularly useful for the study of weak interactions and for the screening of binding ligands with atomic resolution.

## 1 Introduction

Interactions of proteins with other molecules essentially define their functions.^1^ Such interactions are not only involved in almost every process in biological systems, but are also key events, when the external modulation of protein function by drugs is desired.^2^, ^3^, ^4^ The interaction partner span a wide range from ions, small molecules, lipids, peptides to other proteins or membranes. Nuclear magnetic resonance (NMR) spectroscopy is a very efficient technique in order to get information about protein–ligand interactions at atomic resolution. Besides providing structural information, it also allows for a fast screening, especially of weakly binding ligands. There are several approaches available, which can be used to study protein ligand interactions by solution NMR spectroscopy. The methods can be primarily categorized into protein observed or ligand observed techniques. In a protein observed method, a spectrum of protein is acquired and the ligand is titrated. This provides information about the residues in the protein, which are involved in the direct interaction with the ligand. In ligand observed methods a spectrum of the ligand is acquired and the protein is added. The ligand can be anything ranging from a small molecule like a chemical compound or a peptide to a macromolecule like DNA or another interacting protein. The equilibrium involved in a single two side protein (A)–ligand (B) complex formation A+B⇔AB is described by the dissociation constant *K*_d_ 5

(1) $$K_{d}=\frac{\left[A\right]\left[B\right]}{\left[AB\right]}$$

where [A], [B], and [AB] are equilibration concentrations of the reactants A and B and the complex AB, respectively. For a bimolecular interaction the units of thermodynamic equilibrium constant *K*_d_ are in molar concentrations. Strongest binding in biomolecular complexes has been found on the order of 10^−15^ m for biotin binding to avidin,^6^ and on the extreme, a physiologically active protein protein interaction in phosphorylation signaling with a *K*_d_ of 25 mm has been reported.^7^ The size of *K*_d_ is determined by the on (*k*_on_) and off (*k*_off_) rates of the ligand on its target according to

(2) $$K_{d}=\frac{k_{off}}{k_{on}}$$

The lifetime of the protein–ligand complex, which is given by 1/*k*_off_ is responsible for the overall appearance of the NMR spectra. Two limiting cases can be described: slow and fast chemical exchange between the free and protein‐bound form of the ligand. In the slow exchange regime the lifetime of the protein–ligand complex is much longer than the difference in chemical shifts between two signals observed for the free (*ω*_f_) and bound (*ω*_b_) form that is, |*ω*_f_−*ω*_b_|≫1/*k*_off_. This situation, which is found typically for strong complexes results in two observable NMR signals. On the other side a weak binding event is found when |*ω*_f_−*ω*_b_|≪1/*k*_off_. In this case the ligand exchanges fast between the free and bound form, which leads to a collapse of the two signals into a single peak, as the lifetime is too short for the observation of individual signals on the NMR timescale. Some of the NMR techniques used for investigating protein–ligand interactions only work in the fast exchange regime, while others are only possible for strongly interacting molecules in slow exchange. Their respective windows of interaction strength are discussed for the individual methods. Besides the exchange regime, the size of the protein and the ligand and of course the desired information have to be considered in selecting the most appropriate NMR experiment for a particular interaction.

## 2 Protein‐Based Methods

### 2.1 Chemical Shift Mapping

**Figure 1:**
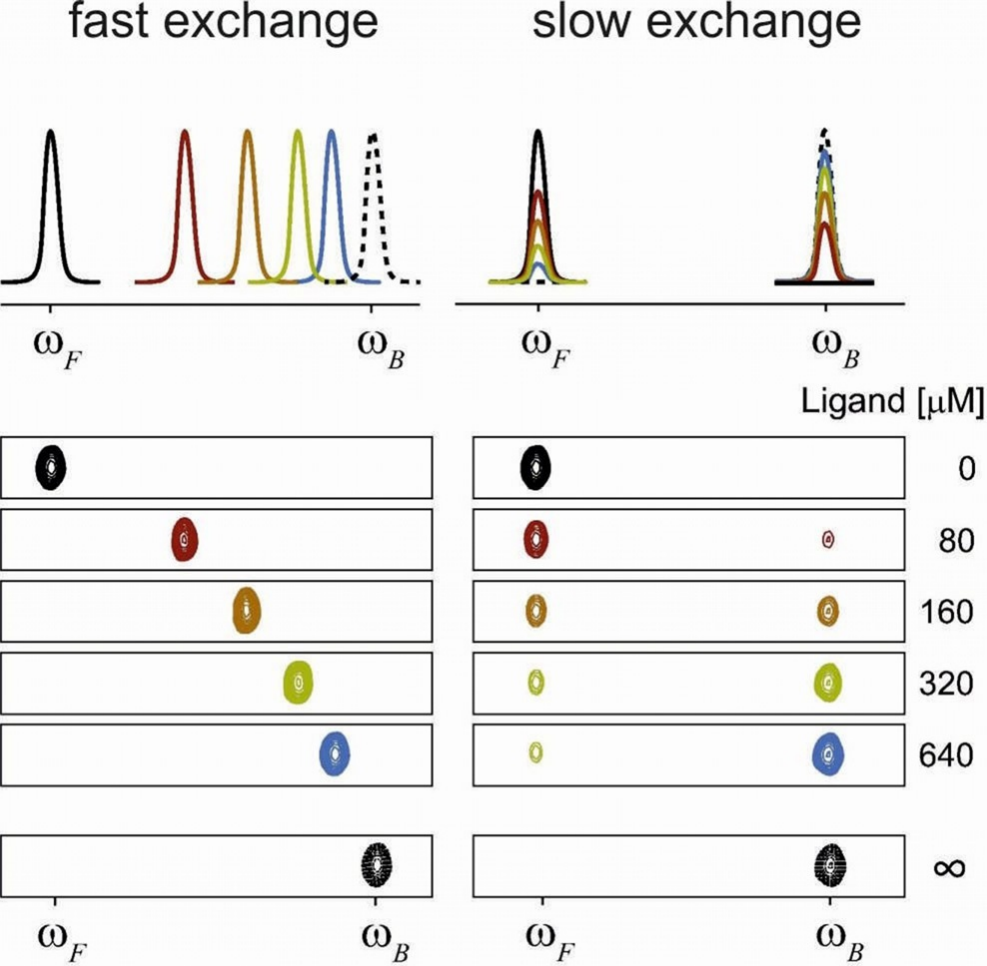
Changes of protein peaks upon titration with a ligand are shown schematically for fast and slow exchange. For binding in fast exchange, the signal of the free‐protein peak at *ω*_F_ is moving towards the fully ligand‐saturated protein peak *ω*_B_. In slow exchange, only the relative signal intensities of free and bound protein peaks change. Adapted from Ref. 2 with permission.

Upon formation of protein–ligand interactions several physical parameters of both the protein and the ligand change. First of all there will be changes in the local electron density due to for example, differences in the hydrophobicity at the interaction surface. Differences in the electron density have an influence on the most easily observable NMR parameter‐ the chemical shift. Large changes in chemical shifts are also induced by the spatial proximity of groups with magnetic susceptibility anisotropies, like aromatic rings. Importantly, the chemical shift is not only influenced by the change in the covalent molecular structure of a protein but also by the non‐covalent interactions with ligands and solvent molecules. One of the most important protein observed methods for the investigation of protein–ligand interactions is the chemical shift mapping (CSM) also known as the chemical shift perturbation (CSP) or complexation induced changes in chemical shifts (CIS).^8^ Thereby, a series of NMR spectra of the protein are recorded in the absence and presence of varying amounts of the binding ligand. Due to its superior signal dispersion, the most common experiment which is used for chemical shift mapping is the ^15^N‐heteronuclear single quantum correlation (HSQC) experiment. Typically, proteins have to be uniformly labeled with ^15^N by producing them in genetically engineered *E. coli* bacteria. Binding is most easily seen by overlaying all HSQCs recorded during the titration. If there is an interaction, the chemical shifts of the residues involved in the complex formation with ligand, seen as peaks in a ^15^N‐HSQC, are displaced from their original position. As described above, two limiting cases are found. In the fast exchange limit the two signals collapse to one, whose chemical shift represents the population averaged value of the free and ligand‐saturated protein. Depending on the relative amount of protein, ligand and the *K*_d_ value the resulting signal is somewhere between the free and bound state. In the slow exchange regime both signals of bound and free state are observed with signal integrals representing their relative amounts (see Figure 1).

**Figure 2:**
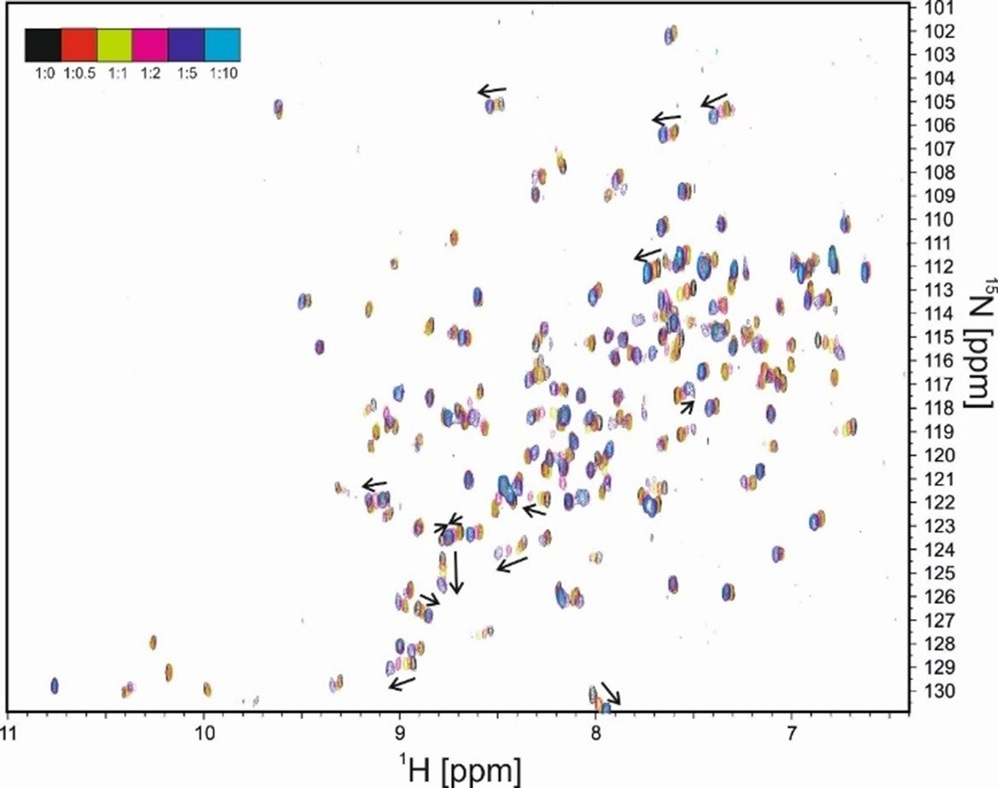
Overlap of six ^1^H–^15^N HSQC spectra of hen egg white lysozyme, titrated with increasing amounts of histamine. The ratios of lysozyme to histamine and the color code of each spectrum are indicated in the top left corner.

As an example of chemical shift mapping, a titration of lysozyme with histamine^9^ is shown in Figure 2. For this titration, a series of ^15^N‐HSQC spectra was acquired on natural abundance hen egg white lysozyme at a concentration of 5 mm with 32 scans per increment, amounting to a total experimental time of just over 1 hour for each two‐dimensional spectrum. Despite the low natural abundance of ^15^N (0.4 %), reasonable ^15^N‐HSQC spectra can be recorded at such high concentrations.

One essential requirement for chemical shift mapping is that both the protein and the ligand are dissolved in the exact same buffer and measured under the same conditions, since chemical shifts, especially those of amide protons are very sensitive to differences in pH value, temperature and buffer composition. The shifting of a particular signal in CSM experiments does not always indicate that the corresponding residue is close to the binding interface. Conformational changes also lead to differences in resonance frequencies. These peak shifts provide information about allosteric changes in the protein upon the binding of a ligand. There is no direct way to distinguish these shifts from the shifts which result from a direct interaction. However, the peak shifts which are due to a conformational change can be usually observed in a region of the protein target which is buried inside the structure or is located away from the interaction surface. A chemical shift titration can also be used to determine the dissociation constant for weakly bound ligands. The chemical shifts of any affected protein signal measured at different ligand concentrations can be used in a nonlinear least‐square fitting to obtain the *K*_d_ value using the equation below:

(3) $$\Delta \delta_{\mathrm{obs}}=\Delta \delta_{\max}\left[\frac{\left({\left[A\right]}_{t}+{\left[B\right]}_{t}+K_{d}\right)}{2{\left[A\right]}_{t}}\right.\;\;\;\;\;\;\;\left.-\frac{\sqrt{{\left({\left[A\right]}_{t}+{\left[B\right]}_{t}+K_{d}\right)}^{2}-4{\left[A\right]}_{t}{\left[B\right]}_{t}}}{2{\left[A\right]}_{t}}\right]$$

where Δ*δ*_obs_ is the change in the observed shift relative to the free state, Δ*δ*_max_ is the maximum shift change in saturation, [A]_t_ is total protein concentration and [B]_t_ is total ligand concentration. When CSM is carried out on a protein with known resonance assignments, the residues which are involved in the interactions with the ligand are revealed. Chemical shift mapping is also particularly useful for the screening of ligands, and is therefore often used in drug design. It not only provides information about binding and the binding strength, but also about the location on the protein where the interaction takes place. Small ligands that bind weakly to nearby regions can then be synthetically connected in the search of a more tightly binding lead compound in a process called SAR (structure activity relationship) by NMR.^10^

**Figure 3:**
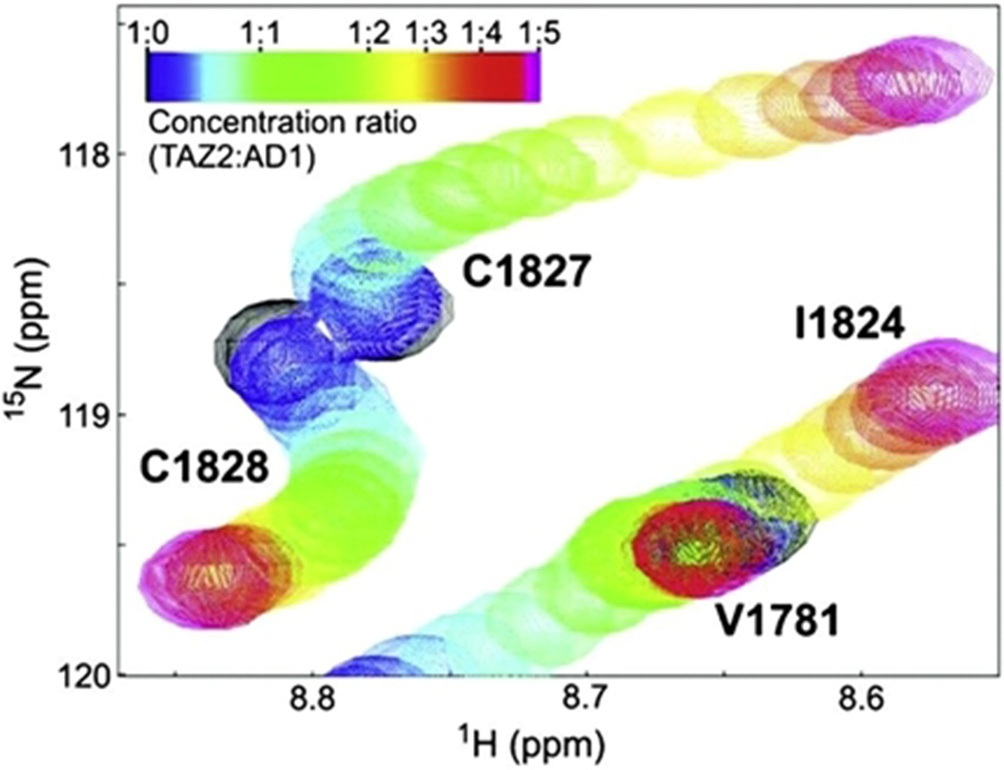
Chemical shift mapping of AD1 binding to TAZ2. Curved lines are indicative of two separate binding events with different *K*_d_ values. Reproduced from Ref. 11 with permission.

In cases where ligand binds to multiple binding sites of a protein with different affinities, a change in the linearity of the HN peak shifting in ^15^N HSQC spectra is observed. The signal shifts in a straight line until the primary stronger binding site is saturated by the ligand and then changes the direction of the shift while the ligand is occupying the second weaker binding site. An example of a nonlinear peak shifting can be seen in Figure 3. The Figure shows a region in the overlaid ^15^N HSQC spectra of the ^15^N labelled TAZ2 domain of a transcriptional coactivator titrated with the unlabeled tumor suppressor p53 domain AD1 domain.^11^ The color of the peaks in the spectrum changes from black to magenta, black indicating the initial spectrum of the free protein and magenta is the final spectrum where the protein to ligand ratio is 1:5. The two binding events were fitted to *K*_d_ values of 24 and 164 μm.

Besides the requirement to use ^15^N isotopically labeled proteins (unless very high concentrations are used), chemical shift mapping relies on well‐resolved protein peaks in the HSQC spectra. The linewidths of the signals increase when going to larger protein due to faster transverse relaxation, resulting from slower molecular tumbling. Well‐structured proteins beyond ≈40–50 kDa typically yield ^15^N‐HSQC spectra whose quality is not good enough for chemical shift mapping. Several methodological developments in the last decades like transverse relaxation‐optimized spectroscopy (TROSY),^12^ deuteration,^13^ stereoarray isotope labeling (SAIL),^14^ direct ^13^C‐detection^15^ or methyl‐TROSY^16^ have expanded the size range of proteins that can be analyzed by solution‐state NMR spectroscopy. However, they often require expensive isotopic labeling strategies. It should also be noted that for intrinsically disordered proteins^17^ much narrower NMR signals^18^ are observed and protein‐protein interactions^19^ can also be investigated on much larger systems.^20^ However, the chemical shift changes upon the interactions of intrinsically disordered proteins are smaller compared to structured ones. Therefore, the preservation of constant solution conditions during the titration is even more important and the high resolution of pure shift spectra might be helpful.^18^, ^21^

**Figure 4:**
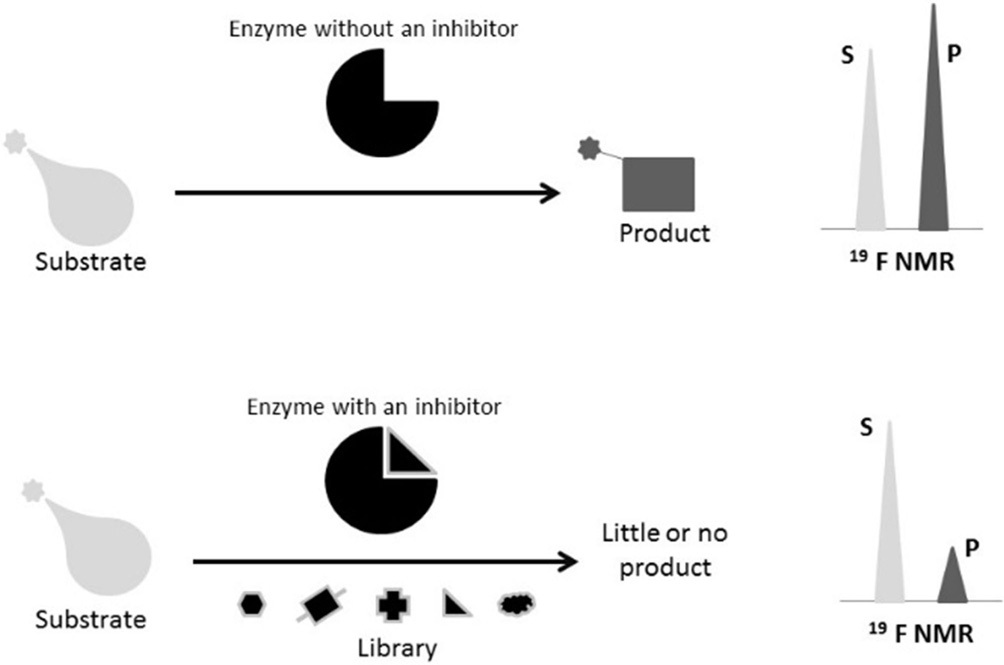
Schematic representation of the FABS method. “S” represents a substrate and “P” a product peak in the ^19^F NMR spectrum. In a sample containing the free enzyme, the fluorine‐containing substrate and product peaks are visible, whereas the presence of an inhibitor suppresses the product peak.

The vast majority of chemical shift mapping experiments is done on ^1^H‐detected spin pairs‐ either with ^15^N or ^13^C. However, ^19^F is a particularly useful nucleus for CSM experiments.^22^, ^23^ It is the only naturally occurring fluorine isotope and the sensitivity is second only to the proton. A ^19^F nucleus is typically shielded by 9 electrons unlike the proton (^1^H), where the nucleus is shielded by one electron. Due to this difference, the range of fluorine chemical shifts (over 400 ppm for organo‐fluorine compounds) and the sensitivity to its environment is much higher when compared to hydrogen. Signal overlap is rarely seen in ^19^F NMR. Naturally occurring proteins do not contain any fluorine nuclei. However, synthetically fluorinated amino acid analogues can be incorporated into proteins.^24^ Incorporation of fluorinated amino acid residue requires a bacterial strain for recombinant protein expression, which is auxotrophic for a given amino acid. For site‐specific labelling, pairs of transfer RNA (tRNA) and aminoacyl‐tRNA synthetase have been developed. An engineered *E.Coli* strain introduced with this pair incorporates the desired synthetic amino acid in vivo during translation.^25^ Another strategy to place fluorine into a protein is in vitro covalent attachment of a fluorinated reactant/fluorinated molecular probe to the functional groups of amino acids. Cysteines are favorable residues due to their ability to form covalent bonds through their side chain sulfhydryl group. However the side chain NH_2_ of lysine or hydroxyl group of serine/threonine can also be used as a possible site of reaction.^24^ In order to investigate protein–ligand interactions by ^19^F NMR a ligand titration using a series of simple 1D fluorine spectra has to be recorded. Fluorine NMR has been also successfully employed for screening potential drug candidates. One indirect way to find enzyme inhibitors is the FABS (fluorine atoms for biochemical screening) approach.^23^ FABS focuses on the substrate conversion of an enzyme to screen for potential inhibitors. In the presence of an active enzyme, both the substrate and the product, which are both fluorine labelled, produce ^19^F signals at distinctive resonance frequencies. When a positive hit for an enzyme inhibitor is present, the ^19^F signal for the product disappears (or becomes less intense). This is because the inhibited enzyme can no longer catalyze the substrate to product or only a little amount of product is formed (see Figure 4).

### 2.2 Hydrogen Exchange

**Figure 5:**
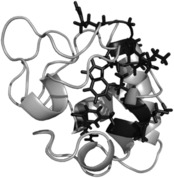
Structure of horse cytochrome c with the residues protected from hydrogen/deuterium exchange in the presence of a monoclonal antibody drawn as line models.

Exchange rates of backbone amide hydrogens with bulk water are often used to get residue‐specific information about the solvent accessibility in a protein. It was proposed more than 40 years ago that changes in amide exchange rates upon the addition of a binding partner could provide an epitope mapping approach for protein–ligand interactions.^26^ Amide protons protected from bulk water by the ligand show reduced exchange rates. Most easily the exchange rates are measured by monitoring signal intensity changes of amide protons upon dissolving the protein in deuterated water. Due to necessary time consuming 2 D NMR acquisitions, only slowly exchanging amide protons can be measured. This approach was first applied to map the binding epitope of a monoclonal antibody to horse cytochrome c.^27^ All residues, whose exchange rates are affected upon antibody binding, were found in a contiguous region on the protein surface of cytochrome c (see Figure 5), indicating no major structural changes upon interaction with the antibody.

Although this method works quite reasonably for stable, well‐structured proteins^27^, ^28^ it should be interpreted with caution when more flexible, less stable proteins are investigated. Binding of the c‐Src SH3 domain to a small peptide ligand led to reduced amide exchange rates throughout the protein. This overall reduction in exchange rates could be attributed to a reduced population of the protein in a high‐energy unfolded state once the ligand is bound.^29^ Hydrogen exchange has been used to study lowly populated high energy conformations as it is a very sensitive probe for such structural changes.^30^ To probe the actual influence of ligand binding on the exchange rates, especially for proteins with a larger proportion of high energy conformations, it is necessary to measure exchange rates at different ligand concentrations.^29^

### 2.3 Solvent Paramagnetic Relaxation Enhancements

Addition of an inert, freely soluble paramagnetic agent to a protein solution leads to increased relaxation of protein nuclei. These solvent paramagnetic relaxation enhancements (sPREs) depend on the distance between the observed nucleus and the paramagnetic probes in the vicinity. The relaxation enhancement (both *T*_1_ and *T*_2_) of a single paramagnetic center are proportional to 1/*r* ^6^,^31^, ^32^ where *r* is the distance between the paramagnetic center and the observed nucleus. For a plane surface the effect of all paramagnetic molecules in solution has to be added up weighted with 1/*r* ^6^, which yields a 1/*d* ^3^ dependence of the sPRE,^32^ where *d* is the distance to the surface. For a protein, whose surface is not really flat, the effect of all paramagnetic centers can be added up by a 1/*r* ^6^ weighted grid search.^33^ Overall, nuclei closer to the surface, that is, more solvent exposed show higher sPREs than nuclei further inside a protein. A bound ligand “protects” the binding interface from enhanced relaxation and can therefore by detected by reduced sPREs. This approach was used for example, for the interaction of matrixmetalloproteinase 3 (MMP3) to tissue inhibitor of metalloproteinase 1 (TIMP‐1). Thereby, sPREs were obtained by monitoring linewidth changes in the presence and absence of the inhibitor, using Gd(EDTA) as the sPRE agent. In this study, broadening of signals could also be observed outside the binding pocket. They could be attributed to conformational changes of the protein upon binding. More quantitative sPREs, obtained from actual relaxation measurements in the absence and presence of the binding partner can be used as experimental input for protein‐protein docking studies.^34^ For exact sPREs the paramagnetic agent needs to be very inert towards the investigated system to prevent locally enhanced sPREs due to specific binding.^35^ Furthermore, only sPREs of non‐exchangeable protons should be used to avoid transfer of very high water sPREs onto the protein.

## 3 Ligand‐based methods

### 3.1 Saturation‐Transfer Difference

**Figure 6:**
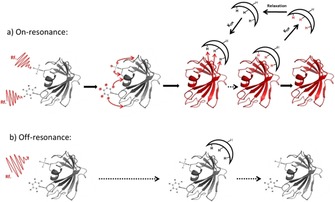
A schematic representation of the STD experiment. Signals of the protein are selectively saturated by RF irradiation. This saturation (indicated in red) is then transferred to the whole protein by spin‐diffusion and further on to the bound ligand, which is in fast exchange with free ligand, where the saturated signals are finally detected.

The saturation‐transfer difference NMR (STD‐NMR) approach is a ligand‐based screening technique, builds upon the nuclear Overhauser effect (NOE)^36^ and works for ligands in fast exchange, with *K*_d_ values in a range of 10^−8^–10^−3^ mol L^−1^. For an STD experiment, the protein target is selectively irradiated by a radiofrequency field which only hits resonances of the protein and removes the magnetic polarization of these nuclei. This is also called the on‐resonance (*I_SAT_*) spectrum, where no ligand resonances are irradiated and frequency values from around −1 to −1.5 ppm are typically chosen. Alternatively, if the ligands do not show resonance signals in the aromatic region, the saturation frequency can be set up further downfield to around 11–12 ppm. When a ligand is in fast exchange between the free and protein‐bound form, the saturation gets transferred through the protein to the bound ligand and by exchange, that saturation is carried on to the free ligand where it is detected with high resolution (Figure 6).

As the method name already implies a second spectrum, where saturation takes place off‐resonance (*I*_0_), has to be acquired. In this experiment the irradiation frequency is set “outside” of any resonance from ligand and protein, for example at 40 ppm and yields a normal spectrum of a mixture. In the difference spectrum (*I*_STD_=*I*_0_−*I*_SAT_) only signals from saturated ligands that interact with the protein will remain. All other components, which do not bind to the protein and consequently are not saturated, will be absent. The saturation through the protein and to the ligand is very fast (on the order of ≈100 ms). Thus, if the off rate of the ligand is fast, the saturation gets quickly into the solution. If a large excess of ligand is used, the saturation of free ligands in solution gets amplified because the relaxation of small molecules is slower than the saturation transfer. The saturation transfer depends mostly on the off rate and therefore, larger off rates produce larger STD signals. However, if the dissociation constant reaches a value of approximately 10 mm, STD signals become very weak as the saturation transfer is not efficient enough. Whenever, compound mixtures become more complex, additional information is needed. In this case, any two‐dimensional NMR experiments can be combined with STD. STD‐NMR is often associated with group epitope mapping (GEM).^37^ Largest signal intensity changes can be found for protons that are in close proximity to their interacting protein. The knowledge about the epitope of the ligand is the starting point for designing and optimizing new drugs.^38^, ^39^ In addition, STD NMR has also been applied to characterize the interactions between ligands in context of membrane protein,^40^ living cells,^41^ viruses^42^ and microtubule assemblies.^43^

### 3.2 Water LOGSY

A variation of STD NMR spectroscopy is WaterLOGSY (water–ligand observation with gradient spectroscopy).^44^ Water plays a crucial role in the protein–ligand, protein‐protein and protein–DNA/RNA interaction mechanism. Water is present at interfaces of interacting molecules.^45^ Water–ligand NOEs are negative, meaning that the residence time of water molcules is longer than 1 ns.^46^ This water can be either squeezed in between ligand and protein or located in a water shell surrounding the ligand. Based on these observations, WaterLOGSY was developed to use bulk water to reveal the binding mechanism of ligands to proteins. By analogy to STD NMR, on‐ and off‐resonance spectra are acquired. The on‐resonance saturation is applied at the water chemical shift and the off‐resonance is applied outside of any ligand/protein resonances. After subtracting the on‐resonance spectra from the off‐resonance spectra, a negative NOE in case of a binding event can be observed. In order to maximize the magnetization transfer rate, WaterLOGSY is using all magnetization transfer pathways such as spin diffusion, etc..^47^ Whenever, the residence time is longer than ≈300 ps the NOEs change sign and increase in magnitude. In general, the bigger the protein the longer the rotational correlation time and consequently, the magnetization transfer is more efficient. The correlation time of the protein can be increased by decreasing the temperature or increasing the solution viscosity.

### 3.3 Cross Saturation/Transferred‐Cross Saturation

**Figure 7:**
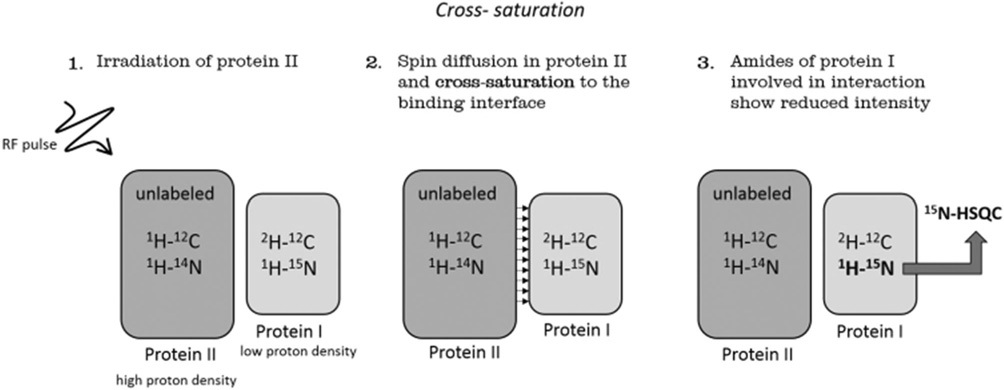
Schematic representation of cross saturation.

Cross‐saturation (CS) is a technique, related to STD, for mapping the binding area between two proteins.^48^ The transferred cross‐saturation (TCS) method is an extension of CS and enables the location of the interface between protein ligands and huge complexes (>150 kDa).^49^ Cross‐saturation requires specific isotopic labeling of one protein. The chosen labeling strategy determines what experiment should be used and which residues are detected. One possible approach is to uniformly label one of the proteins, protein I, with ^2^H and ^15^ n, whereas the other one is unlabeled and as a solvent 10 % H_2_O and 90 % D_2_O is used. Under these conditions protein I has low proton density therefore spin diffusion in protein I is suppressed. The complex is irradiated at a frequency, which only affects protein II, typically aliphatic proton resonances are selected (see Figure 7). Since protein II has a high proton density, spin diffusion takes place and the saturation is immediately transferred to the other protons of protein II and further on to the binding area of protein I via cross‐saturation. There is no spin‐diffusion in protein I, because of the low proton density, so the saturation cannot be transferred further than the interface. Typically, ^1^H‐^15^N HSQC spectra are recorded before and after the irradiation of protein II. Because of the low H_2_O content in the solvent, the amide protons of protein I are partially protonated, so it is possible to detect them but of course with low sensitivity. The residues of protein I, which are at the binding interface can be identified through a reduction in intensity.

**Figure 8:**
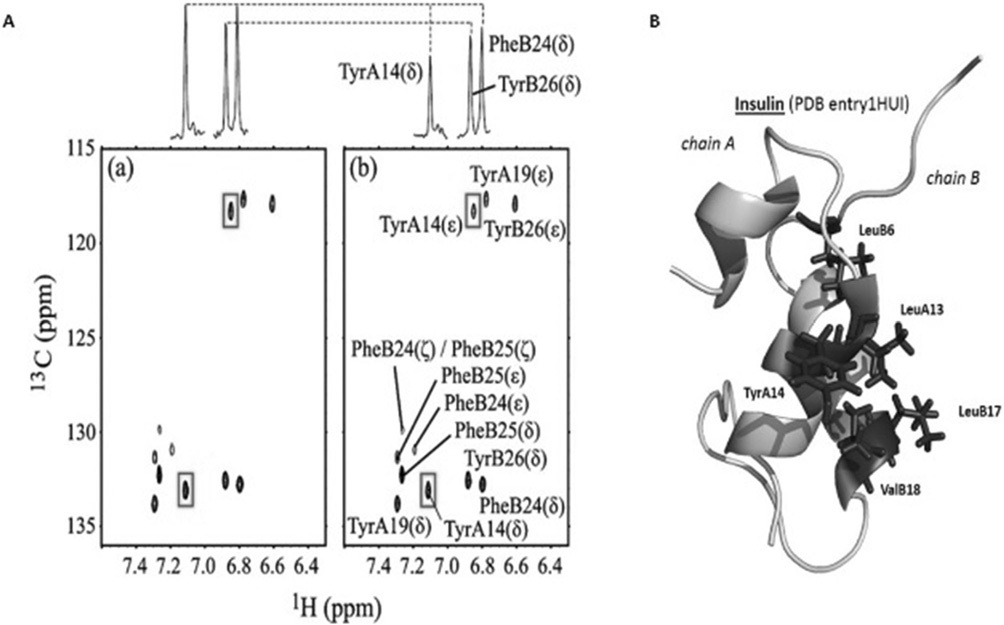
The ^1^H‐^13^C HSQC of insulin before (left) and after (right) irradiation of the insulin receptor. TyrA14 revealed a significant intensity reduction and is therefore directly associated in the binding. B: Structure of insulin (PDB entry 1HUI), indicating the methyl groups and the aromatic residues which show the highest TCS effect. Adapted from Ref. 50 with permission.

The transferred cross saturation (TCS) method is used for huge complexes with low affinity binding. An excess of smaller protein I is used to achieve a fast exchange rate between bound and free state. A reduction in signal intensity can be observed in the free form of the protein I. This technique can be used, for example, for interaction mapping between small soluble and membrane‐attached proteins. As an example of the TCS approach, Shimada and co‐workers used it to characterize the weak binding site of insulin on the insulin receptor (IR).^50^ Transferred CS had to be applied since the IR has a MW of 460 kDa. The used labeling schemes differed slightly from the one described above. Two insulin samples were prepared: one labeled with ^2^H, ^15^N and methyl‐^1^H, ^13^C which was used to detect only the methyl containing residues and the other sample was partially deuterated and ^15^N ^13^C labeled only at aromatic residues. The experiments were carried out in 99 % D_2_O. For studying the IR‐insulin interaction, the ligand insulin was used at an 15:1 excess to achieve fast exchange between the free and bound state. In Figure 8 a zoom of the aromatic HSQCs before and after the irradiation is shown, revealing the intensity reductions of the aromatic residues located in the binding epitope of insulin. All residues of insulin showing signal reductions of at least 30 % through TCS from the insulin receptor are indicated on the structure. They are all exposed on one side of insulin, which contains the binding epitope.

### 3.4 Transferred NOE

**Figure 9:**
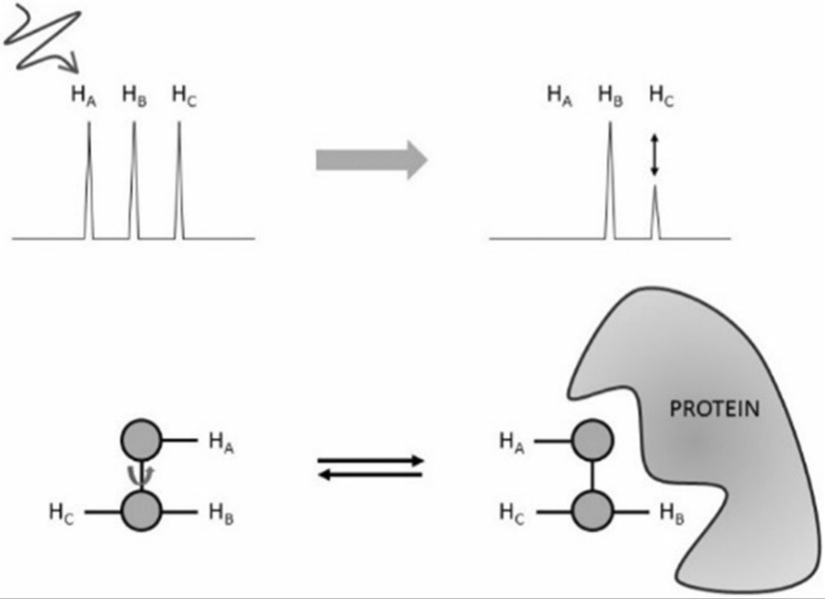
Schematic representation of the transferred NOE experiment. Large negative NOEs build up for a ligand bound to a large protein. When the ligand is in fast exchange between free and bound form, the NOEs are transferred to the free ligand, where they can be observed with high resolution.

The NOE is used for measuring through‐space distances.^51^ It results from a direct dipolar cross‐relaxation between neighboring nuclear spins, which drops off sharply with increasing distance. Usually NOEs between protons can be observed up to ≈5 Å. The sign and size of the NOE depends on the rotational correlation time and therefore on the size of the molecule. Proton–proton NOEs are small and positive for small molecules and large and negative for large molecules. Being a relaxation phenomenon NOEs also take some time to build up and this takes much longer for small compared to large molecules. These properties of the NOE are exploited in transferred NOE (trNOE) experiments.^52^ A small ligand in fast exchange between free and bound to a large protein develops relatively fast a large negative NOE while it is bound to the protein. When it comes off the protein, a small positive NOE forms, but that takes much longer and is therefore rather insignificant. The negative NOE, which is transferred from the bound conformation can be observed in nicely resolved spectra with sharp signals at the position of the free ligand, but at the same time getting important NOE distance information of the ligand in the bound state (see Figure 9).

The NOE could be transferred further on to another competitive ligand, which is observed in the INPHARMA approach.^53^ Thereby, ligands that bind consecutively in the same binding side can be identified by transfer of the NOE from one ligand via the protein to the other ligand.

**Figure 10:**
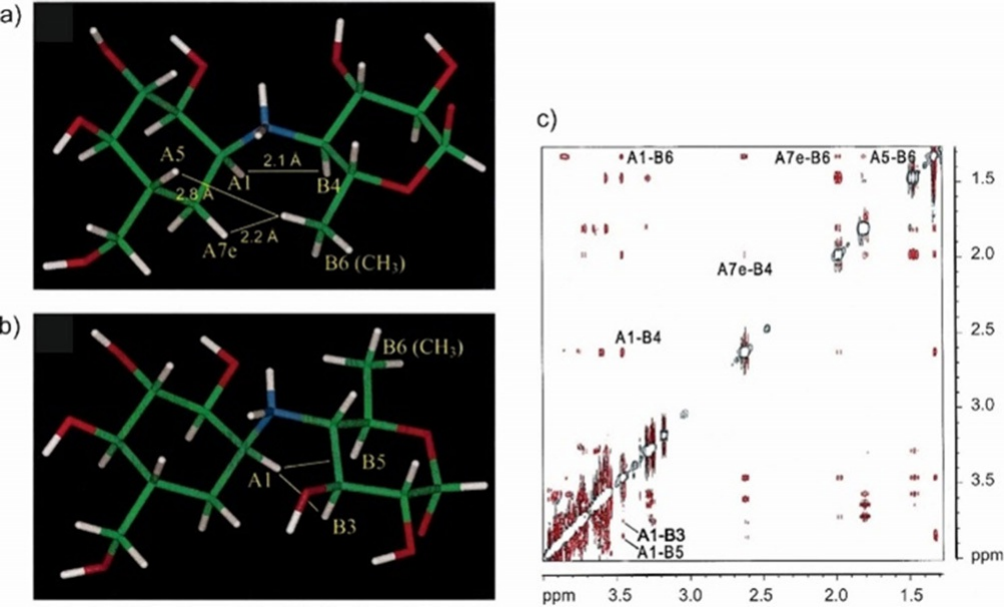
Conformations of GAC1 at the *N*‐glycosidic linkage (a) conformation A found by transferred NOEs (b) “inverted” conformation B, which is found in aqueous solution at the same buffer conditions. In c) the trNOESY spectrum is shown revealing the interglycosidic trNOEs which confirm conformation A in the complex. Reproduced from Ref. 54 with permission.

An example application of the transferred NOE is the binding of d‐gluco‐dihydroacarbose (GAC1), which acts as an inhibitor, to glucoamylase.^54^ The available crystal structure of GAC1 in complex with the catalytic domain of glucoamlyase reveals an unexpected bond conformation of the *N*‐glycosidic linkage. GAC1 has two conformations, which are pH dependent, conformation A is favored under basic conditions (pH 9.0) whereas conformation B is present at low pH (pH 3.0). Although the crystal structure mentioned before was determined under low pH conditions, the bound conformation of the solid‐state complex resembles conformation A. To exclude possible artifacts from crystal packing forces a transferred NOE experiment was used to confirm the unexpected conformation of GAC1, which is selected by the enzyme upon binding. Figure 10 shows both conformations of GAC1 at the *N*‐glycosidic linkage: (a) presents the bound conformation A (b) reveals the inverted conformation B, found in solution. On the right side the trNOE spectrum of GAC1 and glucoamylase at pH 4.5 is shown, revealing interglycosidic trNOEs which confirm conformation A.

### 3.5 NOE Editing/Filtering

**Figure 11:**
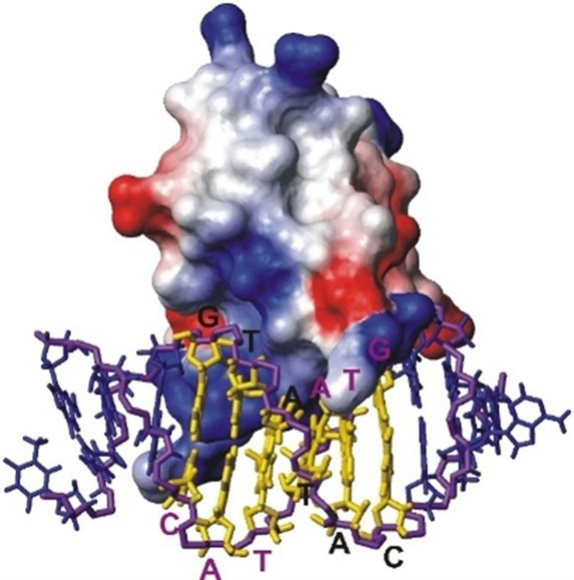
Solution structure of the bacterial antitoxin CcdA bound to its cognate DNA. ^13^C‐edited, ^13^C, ^15^N‐filtered NOESY spectra were used to distinguish intra‐ from intermolecular NOEs.

For tight protein–ligand interactions NOEs can be used for regular structure determinations as long as the size of the complex is within the size limitations of NMR experiments. In order to simplify NOE assignments within the protein, within the ligand and between them, several different isotope labeling (^2^H, ^15^N, ^13^C) schemes are typically used. Combined with isotope editing and filtering NMR experiments one can obtain intramolecular cross‐peaks from either the labelled protein or the unlabeled ligand in the complex, or intermolecular NOEs from their interface region only.^55^ For example, by mixing a ^13^C, ^15^N labeled protein with an unlabeled ligand, one can record intramolecular NOEs of the protein with ^15^N, ^13^C‐edited NOESY spectra. On the other hand, using ^13^C, ^15^N isotope filtering methods the signals of protons bound these nuclei are filtered out, leaving only NOEs of the unlabeled ligand. For detecting intermolecular contacts a ^13^C edited, ^15^N/^13^C filtered NOESY experiments shows only NOEs between ^13^C bound protons of the labeled protein and ^12^C and ^14^N‐bound protons of the ligand.^56^ As an example the NOE‐based structure of the complex between the homodimeric bacterial antitoxin CcdA with its cognate DNA^57^ is shown in Figure 11. Here, intermolecular NOEs were not only essential to determine the binding interface between the protein and DNA, but also to define the even larger interaction region between the two monomers of CcdA. A ^13^C‐edited, ^13^C,^15^N filtered 3 D NOESY‐HSQC was acquired on a sample that contained an equimolar mixture of unlabeled and ^13^C,^15^N‐labeled CcdA. Upon mixing, three different kinds of homodimers form: unlabeled‐unlabeled, labeled‐unlabeled and labeled‐labeled. Using the edited, filtered NOESY only intermolecular NOEs of the 50 % labeled‐unlabeled homodimers are recorded.

### 3.6 Diffusion Editing

**Figure 12:**
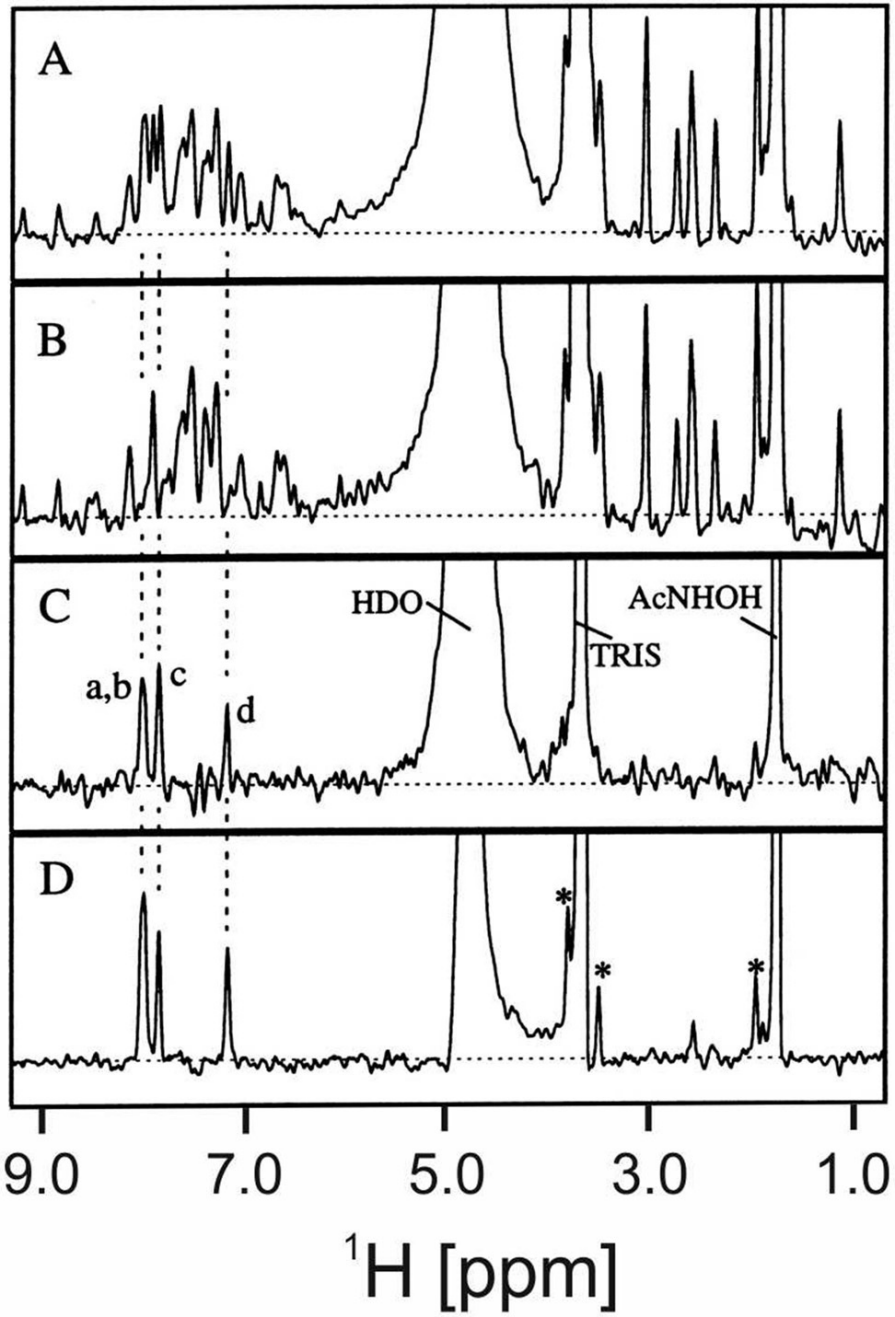
Analysis of ligand binding to stromelysin by using diffusion–editing. A diffusion‐edited spectrum of a mixture of nine compounds in the absence of stromelysin is shown in (A) and in the presence of stromelysin in (B). A difference spectrum, revealing signals of any binding ligand is seen in (C). (D) Reference spectrum of 4‐cyano‐4′ hydroxybiphenyl alone. Signals from impurities in the buffer are indicated by asterisks. Adapted from Ref. 59 with permission.

The diffusion behavior of small molecules is significantly different from the one of large biomolecules, since diffusion coefficients are inversely proportional to the hydrodynamic radius. Translational diffusion can be measured by NMR spectroscopy quite conveniently using pulsed field gradients (PFG), which produce a linear magnetic field variation across the NMR sample. The precession frequency depends on the overall magnetic field. A PFG leads to varying precession frequencies for a particular signal across the NMR sample and therefore defocusing of its magnetization. Refocusing is achieved by applying another PFG for exactely the same duration and with the same strength after inverting the magnetization with a 180° radio frequency pulse. This refocusing into observable magnetization only works if there is no diffusion in the NMR sample. Translational diffusion results in a decay of the magnetization according to

(4) $$I=I_{0}e^{-D{\left(\gamma \delta G\right)}^{2}\left(\Delta -\frac{\delta }{3}\right)}$$

where *I* is the observed intensity after the application of the two gradients, *I*_0_ the intensity with zero gradient strength, *D* the diffusion coefficient, *γ* the gyromagnetic ratio, *δ* the duration of the gradient with strength *G* and *Δ* the time between the two gradients. The diffusion coefficient of the ligand in equilibrium between free and protein‐bound form is given by:^58^

(5) $$D_{e}=D_{f}\frac{A_{f}}{A_{T}}+D_{b}\frac{A_{b}}{A_{T}}$$

where *D*_e_ is the experimentally determined diffusion coefficient of the ligand, *D*_f_ the one of the free ligand and *D*_b_ the ligand bound to the protein (so for small ligands the one of the protein), *A*_b_ and *A*_f_ are the mole fraction of bound and free ligand, respectively, and *A*_T_ is the total amount of ligand in solution (*A*_T_=*A*_b_+*A*_f_). Fast diffusing small molecules can be removed from the spectrum through their faster decay in a diffusion filter. Compounds that bind to a large biomolecule show significantly slower diffusion and remain in the spectrum. This approach, which has also been called “affinity NMR” allows the screening of small molecule libraries for compounds to bind to a macromolecule. As an example, the binding of 4‐cyano‐4′ hydroxybiphenyl to stromelysin^59^ is shown in Figure 12.

Diffusion editing only works if there is a significant size difference between the protein and ligand since the hydrodynamic radius is proportional to the cubic root of the molecular weight (MW^1/3^).

### 3.7 Relaxation Editing

Besides translational diffusion, rotational tumbling also depends on the molecular size. A large biomolecule has a much longer rotational correlation time than a small molecule. This leads to shorter *T*_2_ relaxation times (broader lines) for larger molecules, whereas *T*_1_ relaxation times can be comparable. Due to different relaxation mechanisms this effect is even stronger for ^19^F as compared to ^1^H. Binding of small molecules can be observed through line‐width changes, upon the addition of a binding protein.^2^, ^60^ Dissociation constants can be obtained by monitoring the linewidth or signal intensities as a function of binding partner concentrations. Instead of the linewidth, the actual *T*_2_ relaxation times can be followed, or to avoid problems due to scalar coupling evolution, *T*_1_ *ρ* relaxation times are frequently used. *T*_1_ *ρ* relaxation is active during a spin‐lock field, which supresses the evolution of homonuclear scalar coupling. Relaxation‐editing is achieved typically using a Carr—Purcell—Meiboom–Gill (CPMG) sequence, which allows for *T*_2_ relaxation time measurements by following the signal decay as a function of the relaxation delay. Setting this delay to several hundreds of ms removes fast relaxing signals from the spectrum. To screen for binding ligands relaxation‐edited spectra in the absence of presence of the protein have to be recorded. Bound ligands are removed from the relaxation‐edited spectrum in the presence of protein and show up after subtraction of the two spectra. A wide range of binding affinities can be investigated by relaxation‐editing. Typically short CPMG times will be used for high affinity ligands, since their relaxation is more strongly influenced upon binding, while longer relaxation delays have to be used for weakly binding ligands.

### 3.8 Paramagnetic Tags

Spin labels, specifically organic radicals or paramagnetic lanthanide based labels have a long history in NMR and have been used as chemical shift mediators and line broadening agents to determine conformational and structural changes in proteins since the 1970s.^61^ A paramagnetic center in a protein can be used to probe for ligand binding in its vicinity. Unpaired electrons of paramagnetic probes cause an increase of relaxation rates of nuclei up to a distance of about 20 Å. This phenomenon is known as paramagnetic relaxation enhancement (PRE) and causes line broadening in the spectra. The magnitude of the PRE depends on the square of the gyromagnetic ratio, the inverse sixth power of the interspin distance and the correlation time and can be described by the transverse relaxation rate enhancement *R*_2_ [Eq. (6)].^62^

(6) $$R_{2}=\;\frac{1}{15}\;S\left(S+1\right)\frac{\gamma_{I}^{2}\;g^{2}\;\beta^{2}\;}{r^{6}}\left(4\tau_{c}+\;\frac{3\tau_{c}}{1+\omega_{I}^{2}\tau_{c}^{2}}\right)$$

S is the electron spin, *γ*_I_ the proton gyromagnetic ratio, g the electron g factor, *β* the Bohr magneton, *r* the distance between the electron spin and the nuclear spin, *ω*_I_ the resonance frequency of protons, and *τ*_c_ the correlation time of the vector connecting the electron and nuclear spins. The correlation time *τ*_c_ can be described by Equation (7) and depends on the rotational correlation time of the protein–ligand complex *τ*_r_, the electronic relaxation time *τ*_s_ and the lifetime of the complex *τ*_m_.

(7) $$\frac{1}{\tau_{c}}=\;\frac{1}{\tau_{r}}+\;\frac{1}{\tau_{s}}+\frac{1}{\tau_{m}}$$

All lanthanides have similar chemical behavior and are inert. Diamagnetic control samples can be easily prepared using different lanthanides. Strong paramagnetism is observed for Dy^3+^, Tb^3+^, Tm^3+^, and moderate paramagnetism in Er^3+^, Ho^3+^, Yb^3+^, whereas La^3+^, and Lu^3+^ are diamagnetic. The vast majority of natural protein do not contain a paramagnetic center. However, several approaches are available to introduce a paramagnetic probe into a protein. One way is to bind a synthetic paramagnetic probe site‐specifically onto a protein surface via reactive amino acids, such as cysteine or artificial amino acids. Other cysteines, which might be present in the protein would need to be mutated away. However, cysteine mutations are often found to destabilize the protein fold and some cysteines are essential for the protein function. As an alternative it is possible to employ non‐natural amino acids for site‐specific labeling of proteins.^63^ For instance a paramagnetic nitroxyl center can be attached to *p*‐azido‐l‐phenylalanine by click chemistry.^64^ Metal‐containing proteins offer another approach to paramagnetic labelling. A naturally bound, non paramagnetic metal in a protein could be replaced with a paramagnetic metal ion. This strategy has been successfully used to determine the structure of the 30 kDa exonuclease domain *ϵ* of *E.coli* DNA polymerase III (Pol III) in complex with another Pol III subdomain, *θ*.^65^ It is also possible to use a lanthanide binding peptide (LBP) which can be genetically engineered into the protein. This approach was used for the screening of low‐ and high affinity ligands of the Src homology 2 (SH2) domain of growth factor receptor‐bound protein 2 (Grb2).^66^ Binding of ligands to a paramagnetically tagged protein can be identified using regular relaxation‐editing approaches.^67^ With paramagnetic tags on a protein, especially when several of them are used at different locations on the protein surface it is possible to determine the site and orientation of the ligand on the protein. Ligand signals closer to the paramagnetic center experience larger relaxation enhancements compared to the ones on the opposite side of the ligand.^68^

### 3.9 Residual Dipolar Couplings

When molecules are partially aligned in a magnetic field, an additional splitting can be observed which depends on the orientation of the bond between the coupled spin pair relative to the magnetic field. The reason for this splitting is the dipolar coupling, a direct interaction between the nuclear magnetic moments, which is very strong in the solid state, leading to very broad signals but averages to zero in isotropic solution. Several means are available to produce small degrees of ordering, like bicelles, bacteriophages, polymer gels or the inherent anisotropy of the magnetic susceptibility of some molecules.^69^, ^70^ The resulting residual dipolar couplings (RDCs) provide structural information, which is complementary to the distance information provided by the NOE.^70^ In favourable cases the mode of binding of a ligand to a protein can be determined by the measurement of RDCs of the ligand in the presence of the protein in an alignment medium. Since the RDC also depends on the distance between the dipolar coupled nuclei, only one‐bond ^13^C‐^1^H and ^15^N‐^1^H with known bond‐length are used. Therefore, the ligand has to be labelled isotopically when using RDC information. The first application of RDCs in protein–ligand interactions was carried out by Prestegard and co‐workers.^71^ They observed residual dipolar couplings of ^1^H‐^13^C spin pairs in α‐methyl mannoside in the presence of mannose‐binding protein‐A (MBP) in a field‐oriented aqueous liquid crystal. Generally much smaller splittlings were observed for α‐methyl mannoside in the same alignment medium without MBP, but they are not scaled down and vary from site to site, indicating a different orientation of free and MBP bound ligand. Since α‐methyl mannoside is a weak binding ligand, the observed RDCs represent a population weighted average of those in the free and bound form. RDCs originating from the bound state can then be calculated with the known dissociation constant. Using the known structure of α‐methyl mannoside and MBP a binding mode of this complex could be deduced from five experimental RDCs with singular value decomposition.^72^ In favourable cases, residual dipolar couplings can also be used when the protein target is very large or even embedded in an oriented membrane bilayer. An example is the transient binding of the C‐terminal transducing undecapeptide, which was selectively ^15^N‐labelled at Leu‐5 and Gly‐9.^73^ Fast exchange between the free and bound form of the peptide enabled its partial alignment and measureable RDCs.

## 4 Summary and Outlook

Protein–ligand interactions play a pivotal role in almost all process in biology and for drug development. Their elucidation is often more important for understanding the function(s) of a protein than its 3 D structure. NMR spectroscopy is at the forefront of methods for investigating protein–ligand interactions as it provides a plethora of technique to reveal with atomic resolution the interaction mechanisms for both weakly and tightly bound ligands. The information provided could be anywhere from the simple confirmation of binding to the 3 D structure of the complex. Although some methods, like the transferred nuclear Overhauser effect (NOE) or saturation‐transfer difference (STD) can provide information even for extremely large complexes, protein‐derived information is still constrained by the NMR size limit, rendering structural information of proteins beyond ≈50 kDa often impossible to obtain. Protein–ligand investigations by NMR are inherently in vitro experiments. NMR solution studies on such interactions in vivo, in living cells are just beginning to emerge^74^ and could open new and exciting ways for future studies.

## Conflict of interest

*The authors declare no conflict of interest*.

## Biographical Information

Walter Becker is a Ph.D. candidate at the University of Graz, Institute of Chemistry. He received his MSc. in Biochemistry and Molecular Biomedicine at the TU Graz in 2016. Part of his Ph.D. work was carried out at the Australian National University in Prof. Otting's lab. His research interests include the structural biology of membrane proteins and the development of new “NMR active” tags.

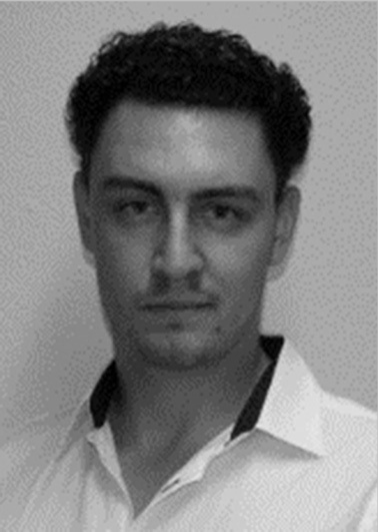

## Biographical Information

Krishna Chaitanya Bhattiprolu received his bachelors in technology (B.Tech) in Biotechnology from Andhra University (India) in 2010 and Master of Science (M.Sc.) in Applied Biotechnology from Uppsala University (Sweden) in 2012. Since 2014 he is a Ph.D. candidate at the University of Graz, Institute of Chemistry. His research interests include the structural and functional characteristics of intrinsically disordered proteins.

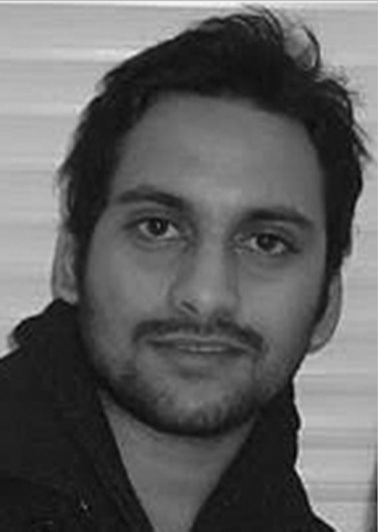

## Biographical Information

Nina Gubensäk received her B.Sc. and M.Sc. degrees in Molecular Biology and Biochemistry at the University of Graz in 2011 and 2014, respectively. In 2016, she was granted a DOC Fellowship by the Austrian Academy of Sciences and is currently a Ph.D. candidate at the University of Graz. Her scientific interests lie in the field of structure determination using solution NMR as well as NMR method development.

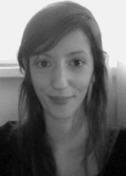

## Biographical Information

Klaus Zangger received his Ph.D. degree in chemistry in 1996 at the University of Graz. After postdoctoral work in the lab of Ian M. Armitage at the University of Minnesota(USA), he returned to the University of Graz in 1999 to the Institute of Pharmaceutical Chemistry. He received an Associate Professor position at the Institute of Chemistry in 2003. His scientific interests are in the field of solution NMR of biomolecules and methods’ development.